# Effect of Cyclodextrin Complexation on the Separation of Insulin Analogues in Capillary Electrophoresis Using Dynamically Coated Capillaries

**DOI:** 10.3390/molecules31193578

**Published:** 2026-10-08

**Authors:** Csenge Kis, Emese Sipos, Gergő Tóth, Zoltán-István Szabó

**Affiliations:** 1 Department of Physical Chemistry, “George Emil Palade” University of Medicine, Pharmacy, Science, and Technology of Targu Mures, 540142 Târgu Mureş, Romania; 2 Doctoral School of Medicine and Pharmacy, “George Emil Palade” University of Medicine, Pharmacy, Science, and Technology of Targu Mures, 540142 Târgu Mureş, Romania; 3 Department of Pharmaceutical Industry and Biotechnology, “George Emil Palade” University of Medicine, Pharmacy, Science, and Technology of Targu Mures, 540142 Târgu Mureş, Romania; 4 Department of Pharmaceutical Chemistry, Semmelweis University, Hőgyes E. u. 9, 1092 Budapest, Hungary; 5 Center for Pharmacology and Drug Research & Development, Semmelweis University, 1092 Budapest, Hungary; 6 Sz-imfidum Ltd., 525401 Lunga, Romania

**Keywords:** insulin analogues, capillary zone electrophoresis, cyclodextrins, coated capillaries

## Abstract

The simultaneous separation of therapeutic insulin analogues by capillary electrophoresis (CE) remains challenging because of their high structural similarity and their tendency to adsorb onto the capillary surface. This study investigated the applicability of cyclodextrin (CD) derivatives as selectivity-modifying additives for the CE separation of human insulin and five therapeutic insulin analogues using five-layer poly(diallyldimethylammonium chloride)/poly(sodium 4-styrenesulfonate) (PDADMAC/PSS)_2.5_ and four-layer (PDADMAC/PSS)_2_ successive multiple ionic-polymer layer (SMIL)-coated capillaries under acidic and alkaline conditions, respectively. A broad range of CD derivatives was systematically evaluated under optimized background electrolyte conditions. Under acidic conditions, neutral and predominantly uncharged CD derivatives improved separation selectivity, with carboxyethyl-β-CD providing the most favorable discrimination within the critical insulin lispro–human insulin–insulin aspart migration group. In contrast, highly charged anionic CD derivatives disrupted the PDADMAC-terminated coating and could not be applied. The PSS-terminated coating remained stable under alkaline conditions, enabling the evaluation of both neutral and anionic CD derivatives. Under these conditions, the migration order of the insulin species was completely reversed, while several CD derivatives further modulated the relative spacing of neighboring peaks. The use of organic modifiers produced system-dependent effects on separation selectivity and peak shape. These findings demonstrate that the appropriate selection of CD derivatives and SMIL coating strategy provides an effective approach for modulating selectivity in the CE separation of structurally related therapeutic peptides.

## 1. Introduction

Human insulin is a peptide hormone secreted by the β-cells of the pancreatic islets of Langerhans. Structurally, it consists of two polypeptide chains, designated A and B, linked by two interchain disulfide bonds, while an additional intrachain disulfide bond contributes to the structural stability of the A chain. In total, insulin contains 51 amino acid residues distributed between the 21-residue A chain and the 30-residue B chain [1]. The primary physiological function of insulin is the maintenance of glucose homeostasis through the stimulation of glucose uptake in peripheral tissues, particularly skeletal muscle and adipose tissue, and the suppression of hepatic glucose production [2]. Impaired insulin secretion, impaired insulin action, or a combination of both contributes to the pathogenesis of diabetes mellitus, predominantly type 1 and type 2 diabetes mellitus [3]. Although recombinant human insulin represented a major therapeutic advancement, currently available insulin formulations are unable to fully replicate the physiological pattern of endogenous insulin secretion. Consequently, structural modifications involving minor alterations in the amino acid sequence of human insulin have been introduced to improve its pharmacokinetic and pharmacodynamic characteristics, leading to the development of long-acting and rapid-acting analogues [4].

The analytical separation and quantification of human insulin and its therapeutic analogues present a considerable challenge due to their high structural similarity. In most cases, insulin analogues differ from native human insulin only by one or two amino acid substitutions or by minor chemical modifications [5]. Despite these difficulties, a variety of analytical approaches for insulin determination have been reported in the literature and can be classified into immunological and separation techniques. Immunological techniques, including enzyme-linked immunosorbent assay (ELISA), amplified luminescent proximity homogeneous assay (AlphaLISA), homogeneous time-resolved fluorescence (HTRF), chemiluminescent assay (CLIA), radioimmunoassay (RIA), and on-chip immunoassays, are widely used because of their high sensitivity and suitability for routine analysis. However, these methods often lack sufficient selectivity for the discrimination of individual insulin analogues, as antibody cross-reactivity frequently occurs among these species [6].

Separation techniques are more suitable for the differentiation of human insulin and its therapeutic analogues. Among these, high-performance liquid chromatography (HPLC) methods are the most widely employed analytical methods for insulin determination. In the current European Pharmacopoeia, the identification and quantification of human insulin and its analogues are primarily based on HPLC methods coupled with UV detection [7,8,9,10]. Recent studies have described advanced chromatographic approaches capable of the simultaneous separation of multiple therapeutic insulin analogues, as well as their deamidated forms, variants, and aggregates, even in complex matrices with high sensitivity [11,12,13,14,15,16].

Although HPLC is widely used for insulin analysis, capillary electrophoresis (CE) offers complementary selectivity for closely related insulin analogues, together with high separation efficiency, short analysis times, and substantially lower sample and solvent consumption. Numerous CE techniques, such as capillary zone electrophoresis (CZE), micellar electrokinetic chromatography (MEKC), and capillary gel electrophoresis (CGE), have been applied to the analysis of insulin and its analogues. Among these, MEKC and CGE provide particularly high selectivity, especially under alkaline conditions, where insulin molecules carry a net negative charge [5,17,18].

In contrast to MEKC and CGE, CZE is considered one of the most straightforward CE techniques for the analysis of insulin and its analogues. However, interactions frequently occur between insulin molecules and the inner capillary wall, resulting in analyte adsorption, peak broadening, and distorted peak shapes. Consequently, careful optimization of the background electrolyte (BGE) composition, pH, and capillary surface modification is essential for the successful application of CZE to the analysis of insulin analogues [18,19,20,21,22].

One of the most commonly applied strategies for minimizing undesired analyte–capillary wall interactions involves the use of dynamically coated capillaries. Among these approaches, successive multiple ionic-polymer layer (SMIL) coatings have attracted considerable attention. In SMIL-coated capillaries, alternating layers of oppositely charged polymers are deposited onto the inner capillary surface, generating a homogeneous and stable surface charge. By selecting the appropriate outermost polymer layer, the electroosmotic flow can be effectively controlled and separation selectivity can be optimized. Consequently, SMIL-coated capillaries represent a valuable approach for CZE-based separations of proteins and other structurally related biomolecules [23,24].

The aim of this study was to investigate and optimize the CZE separation of human insulin and five therapeutic insulin analogues using SMIL-coated capillaries. To improve the selectivity of the separation, two different SMIL coating systems and a series of neutral and charged cyclodextrin (CD) derivatives were systematically evaluated as separation modifiers. Particular emphasis was placed on investigating the effect of CD complexation on the electrophoretic behavior of structurally closely related insulin species and on identifying the experimental conditions providing optimal resolution.

## 2. Results and Discussion

The electrophoretic separation of insulin analogues remains challenging due to their highly similar primary structures and, consequently, similar electrophoretic mobilities. Under conventional CZE conditions, these small structural differences often result in poor selectivity and limited resolution. An additional challenge in the CE analysis of these peptides arises from their tendency to adsorb onto the inner wall of the fused-silica capillary. This can lead to peak broadening, tailing, reproducibility issues, and loss of separation efficiency.

To overcome these limitations, modification of the inner surface of the capillary is often required. Among these approaches, SMIL coatings have attracted considerable attention. Therefore, the simultaneous separation of human insulin and its five analogues was investigated in CE using two different SMIL-coated capillaries, combined with various BGEs and CDs as additives. Hamidli et al. previously compared bare fused-silica, SMIL-coated, and linear polyacrylamide-coated capillaries for the CE-MS separation of human insulin and its six therapeutic analogues. In agreement with previous observations comparing SMIL-coated and bare fused-silica capillaries, SMIL-coated capillaries outperformed the uncoated counterparts. Nevertheless, problems with peak shape and limited selectivity were also identified [25].

Based on previous reports, several coating strategies were considered using polybrene and poly(diallyldimethylammonium chloride) (PDADMAC) as cationic coating agents and dextran sulfate and poly(sodium 4-styrenesulfonate) (PSS) as anionic coating agents, and a stable and reproducible coating procedure was established using PDADMAC/PSS as coating agents and 0.5 M NaCl as electrolyte. Two types of SMIL-coated capillaries were prepared: a four-layer, (PDADMAC/PSS)_2_-coated capillary, characterized by an anionic outer layer and a cathode-directed EOF, and a five-layer, (PDADMAC/PSS)_2.5_-coated capillary, characterized by a cationic outer layer and an anode-directed EOF. As previous work indicated that acidic conditions provided promising results, the latter SMIL-coating procedure was tested first.

### 2.1. Results Obtained Using the (PDADMAC/PSS)_2.5_-Coated Capillary

#### 2.1.1. Prescreening for an Appropriate Acidic BGE

**Figure 1:**
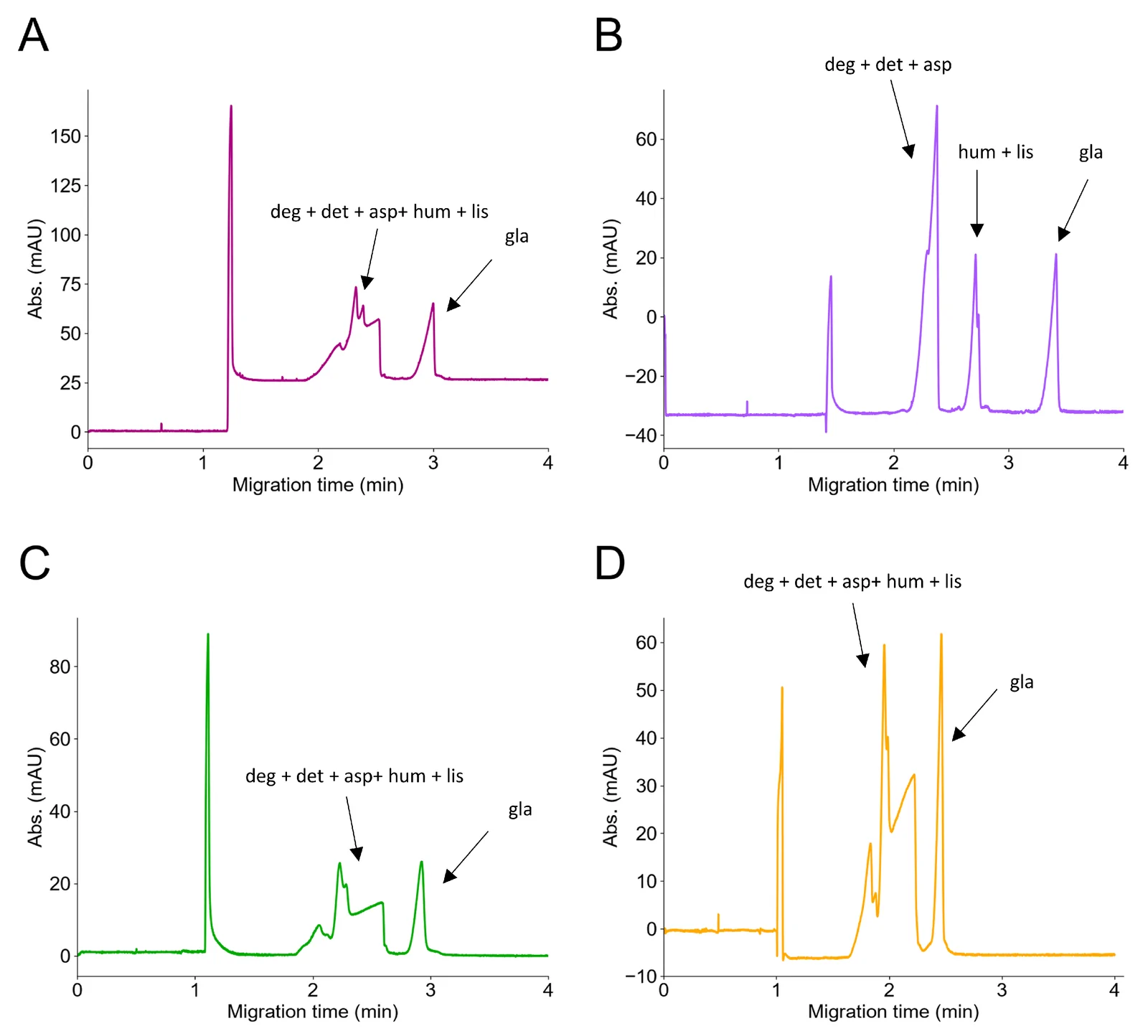
Representative electropherograms obtained using a (PDADMAC/PSS)_2.5_ SMIL-coated capillary with different acidic background electrolytes: (**A**) 1.0 M acetic acid; (**B**) 1.5 M acetic acid; (**C**) 0.5 M formic acid; (**D**) 1.0 M formic acid. Applied voltage: −30 kV; capillary temperature: 25 °C; hydrodynamic injection: 50 mbar × 4 s. Detection at 210 nm.

The cationic outer PDADMAC layer reversed the EOF towards the anode while simultaneously reducing protein adsorption on the inner surface of the capillary under acidic conditions. Among the investigated acidic BGEs (Figure 1), 1.5 M acetic acid provided the most promising electrophoretic performance and was therefore selected for further optimization. Compared with formic acid, acetic acid produced better-resolved peaks while maintaining a stable baseline. Increasing the acetic acid concentration further enhanced the separation selectivity, resulting in narrower peaks and improved resolution, although at the expense of slightly longer migration times. In contrast, formic acid produced broader peaks with partial co-migration of several insulin analogues, indicating inferior selectivity under the investigated conditions. In all cases where it could be determined, the migration order (first to last) was degludec–detemir–aspart–human insulin–lispro–glargine.

#### 2.1.2. CD Screening

The results obtained with the acidic BGEs are consistent with previous reports on the CZE separation of insulin analogues using bare fused-silica and dynamically coated capillaries, where peak distortions were observed with both approaches. Peak tailing was reported with bare fused-silica capillaries, whereas peak fronting was observed with dynamically coated capillaries, particularly for degludec and detemir. In addition, insufficient resolution of the structurally similar insulin analogues was also described [25,26]. Therefore, CDs were subsequently introduced into the BGE as selectivity modifiers. Compared with their widespread application in the chiral separation of small molecules, CDs have been less frequently employed in CZE as selectivity modifiers for peptide separations [27,28]. Nevertheless, several studies have demonstrated that CDs can act as selectivity enhancers for peptides, mainly by modifying electrophoretic mobility through transient complexation and by reducing peptide adsorption to the capillary wall [29,30,31,32,33]. Because insulin molecules are substantially larger than the CD cavity, complete encapsulation is not possible. Instead, the interaction is expected to involve transient and partial inclusion of exposed hydrophobic amino acid side chains, particularly aromatic residues such as phenylalanine and tyrosine, within the hydrophobic cavity of the CDs [34,35,36]. Depending on the nature of the CD derivative, these host–guest interactions may be further complemented by hydrogen bonding and, in the case of charged CDs, electrostatic interactions with ionizable residues located on the protein surface. The combined contribution of these interaction mechanisms may alter the apparent electrophoretic mobility of the insulin species, thereby enhancing separation of the previously co-migrating analytes.

**Figure 2:**
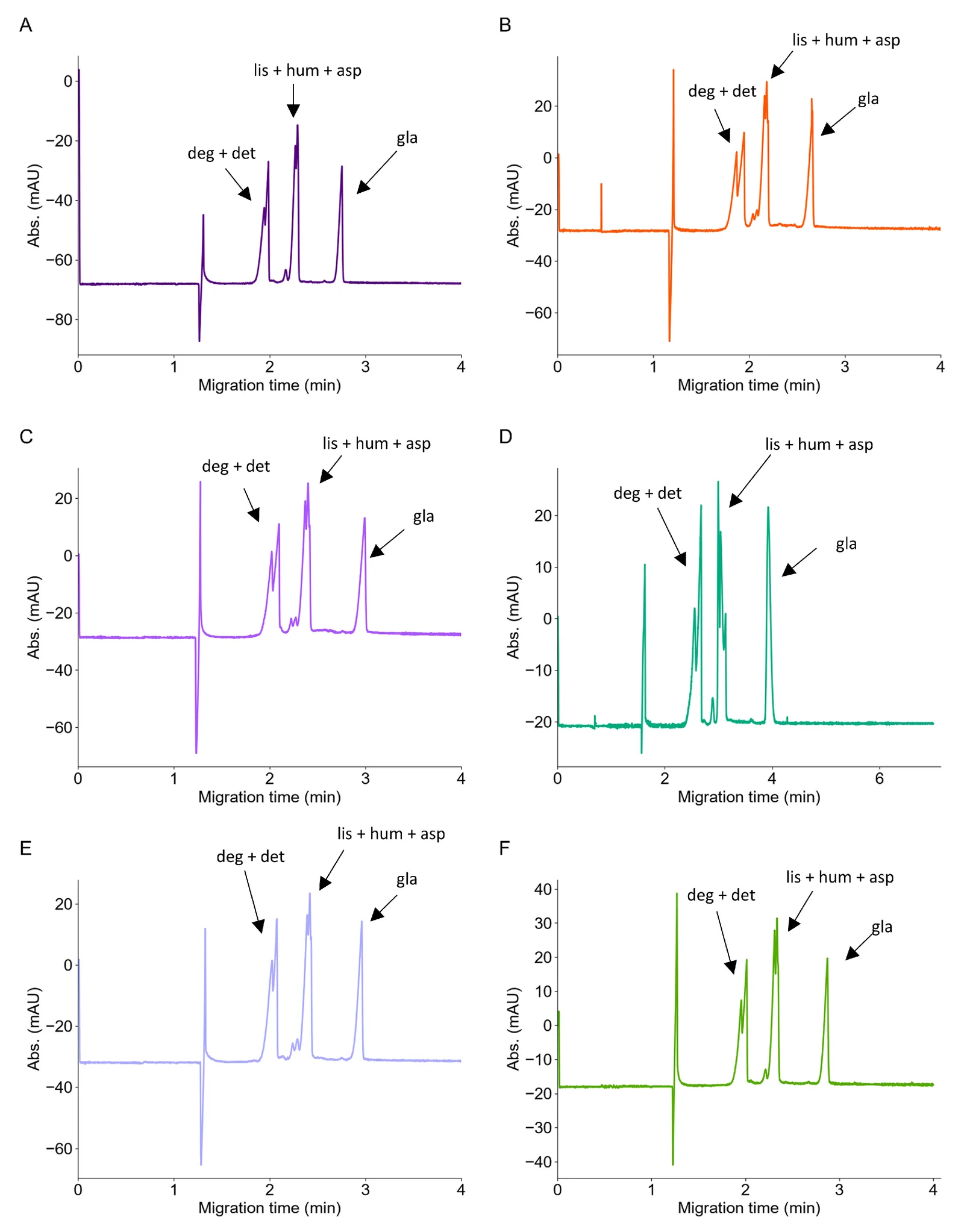
Representative electropherograms obtained using a (PDADMAC/PSS)_2.5_ SMIL-coated capillary with 1.5 M acetic acid BGE and different CD additives: (**A**) 10 mM Ac-β-CD; (**B**) 10 mM α-CD; (**C**) 5 mM β-CD; (**D**) 1 mM CE-β-CD; (**E**) 10 mM DIMEB; (**F**) 5 mM HP-β-CD. Other conditions are the same as in Figure 1.

Following the optimization of the acidic background electrolyte, CDs were introduced into the BGE (the list of the applied CDs is presented in Supplementary Table S1) as selectivity modifiers with the aim of resolving the remaining co-migrating insulin species. The addition of CDs altered the migration behavior of the insulin species and improved peak shapes. Although baseline separation of all six species was not achieved with any of the investigated CDs, all derivatives generally increased selectivity compared with the CD-free electrolyte (Figure 2).

A common feature observed for all investigated CD derivatives was the formation of characteristic migration groups rather than complete separation of the individual insulin species. In most cases, insulin degludec and insulin detemir migrated as the first peak pair, whereas insulin lispro, human insulin, and insulin aspart formed a second unresolved cluster. Insulin glargine consistently migrated as the last and most distinct peak, remaining baseline-separated from the other species irrespective of the CD derivative employed. Although the addition of CDs increased the spacing between these migration groups compared with the CD-free BGE, only relatively small differences in selectivity were observed among the derivatives investigated, resulting in broadly similar separation patterns under the selected conditions.

In the first migration group, insulin degludec and insulin detemir remained only partially resolved and continued to migrate as a partially resolved peak pair. The achievable resolution was further limited by the persistent peak fronting observed for these analogues. Likewise, although the separation between insulin lispro, human insulin, and insulin aspart improved, baseline resolution of this second migration group was not achieved with any of the investigated CD derivatives. These observations suggest that under the selected experimental conditions, the investigated CDs did not induce sufficiently different changes in the apparent electrophoretic mobilities of degludec and detemir, or of lispro, human insulin, and aspart, to achieve baseline resolution. In contrast, glargine remained clearly separated from the other insulin species.

Another characteristic of the separation system was the short analysis time. Owing to the short effective capillary length and the strong EOF towards the anode generated by the SMIL coating, all analytes migrated within 3 min. Compared with the CD-free BGE, the addition of CD derivatives that are predominantly uncharged under the highly acidic separation conditions consistently resulted in shorter migration times for all insulin species. In contrast, increasing the concentration of a given uncharged CD resulted in a slight increase in migration times (representative examples are shown in Supplementary Figure S1).

In contrast to the carboxyalkylated CD derivatives and Succ-β-CD, which are predominantly uncharged under the highly acidic separation conditions, S-β-CD, P-β-CD, and SBE-β-CD retain a high negative charge. Upon addition of these anionic CDs to the BGE, a complete loss of the characteristic anode-directed EOF generated by the SMIL coating was observed, even at sub-millimolar CD concentrations. This behavior suggests that the highly negatively charged CDs interact strongly with the outer PDADMAC layer of the coating, thereby compromising the functional integrity of the coating. As a consequence, these derivatives were unsuitable for further separation experiments. Importantly, the original performance of the coated capillary could be restored after flushing the capillary and reapplying the outer PDADMAC layer, demonstrating functional recovery of the separation system.

Among the investigated CD derivatives, CE-β-CD produced the most promising selectivity enhancement and was therefore selected for further optimization. While the degludec/detemir pair remained only partially resolved, CE-β-CD improved the separation of the second migration group. Unlike the other CD derivatives, which maintained lispro, human insulin, and insulin aspart as a largely unresolved cluster, CE-β-CD induced partial resolution of this cluster, allowing three individual peaks to be distinguished within this region of the electropherogram. Although baseline resolution was not achieved, CE-β-CD provided the greatest selectivity among the investigated CD derivatives for these structurally similar insulin species and was therefore selected for subsequent optimization studies.

#### 2.1.3. Further BGE Optimization: Effect of Combining CDs and Organic Solvents

Since mixed CD systems have often been reported to provide complementary selectivity (especially in chiral CE [37,38,39]), 1 mM CE-β-CD was combined with either α-CD or HP-β-CD. In both cases, the addition of the second CD increased the migration times but reduced the overall selectivity of the separation compared with CE-β-CD alone. Thus, no beneficial complementary selectivity was observed under the investigated conditions, and further optimization was performed using CE-β-CD as the sole CD additive.

The effect of organic modifiers on the separation system was also investigated. Organic solvents are commonly employed in CE to fine-tune separation selectivity by altering the physicochemical properties of the BGE. In particular, they influence the dielectric constant and viscosity of the separation medium, thereby affecting the EOF and the electrophoretic mobilities of the analytes. In CD-mediated separations, organic modifiers may additionally affect analyte–CD interactions by changing the polarity of the medium and the hydrophobic interactions contributing to complex formation. Consequently, the composition of the organic modifier may have a pronounced impact on both migration behavior and separation selectivity.

**Figure 3:**
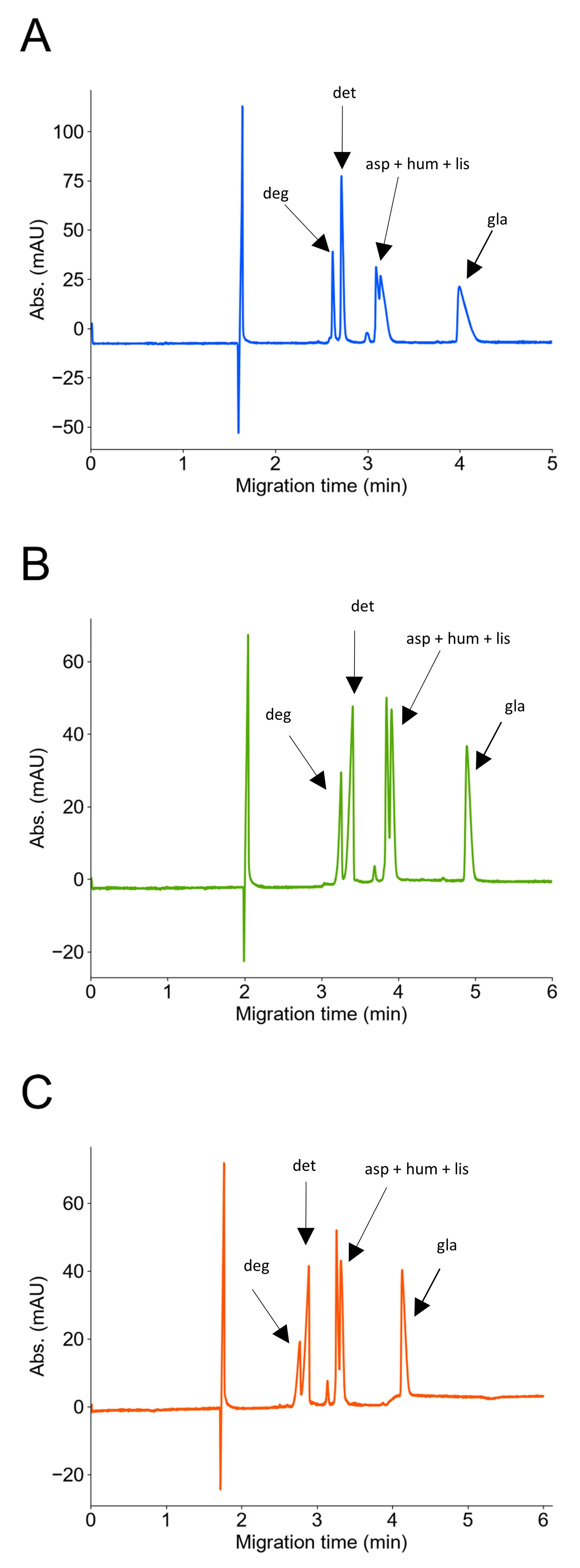
Comparative electropherograms obtained using 10% (*v*/*v*) organic solvents. BGE: 10% (*v*/*v*) (**A**) methanol; (**B**) ethanol; (**C**) acetonitrile: 90% (*v*/*v*) 1 mM CE-β-CD in 1.5 M acetic acid. Other conditions are the same as in Figure 1.

Acetonitrile, methanol, and ethanol were evaluated as organic modifiers at concentrations ranging from 5 to 15% (*v*/*v*) in 5% increments. For all three solvents, increasing the organic modifier content resulted in a gradual improvement in the separation up to 10% (*v*/*v*). However, at higher concentrations, the separation system became unstable, leading to poor run-to-run reproducibility and therefore preventing further optimization in this direction. Comparative electropherograms obtained at 10% (*v*/*v*) organic solvent content are presented in Figure 3.

The most pronounced effect of the organic modifiers was observed for the first migration group, where all three solvents improved the resolution between insulin degludec and insulin detemir. With methanol and ethanol, this peak pair approached baseline separation. In contrast, only modest changes were observed for the second migration group comprising insulin lispro, human insulin, and insulin aspart, which remained incompletely resolved under all investigated conditions.

Although all three organic modifiers improved the separation, distinct differences were observed in their effects on migration behavior and peak shape. Acetonitrile produced the shortest analysis times but provided the lowest overall selectivity among the investigated organic modifiers. In contrast, both methanol and ethanol provided almost baseline separation of the insulin degludec/insulin detemir peak pair. Furthermore, the pronounced peak fronting observed under purely aqueous conditions was substantially reduced, resulting in improved peak shapes.

The principal differences between the two alcohols were observed for the later migrating insulin analogues. While methanol afforded a separation comparable to that obtained with ethanol, it resulted in pronounced peak broadening of the second migration group comprising insulin lispro, human insulin, and insulin aspart, as well as of the insulin glargine peak. This broadening was not observed in the presence of ethanol, which provided the most favorable compromise between separation quality, peak shape, and analysis time.

### 2.2. Results Obtained Using the (PDADMAC/PSS)_2_-Coated Capillary

#### 2.2.1. Prescreening for an Appropriate Alkaline BGE

Since none of the trials led to the baseline separation of all insulin species, further testing was performed using (PDADMAC/PSS)_2_-coated capillaries. In this coating configuration, the outer anionic PSS layer generates a cathode-directed EOF. Under alkaline conditions, the insulin species become negatively charged, which is expected to reduce their interaction with the negatively charged PSS outer layer. In addition, the higher EOF velocity under these conditions can contribute to shorter analysis times.

**Figure 4:**
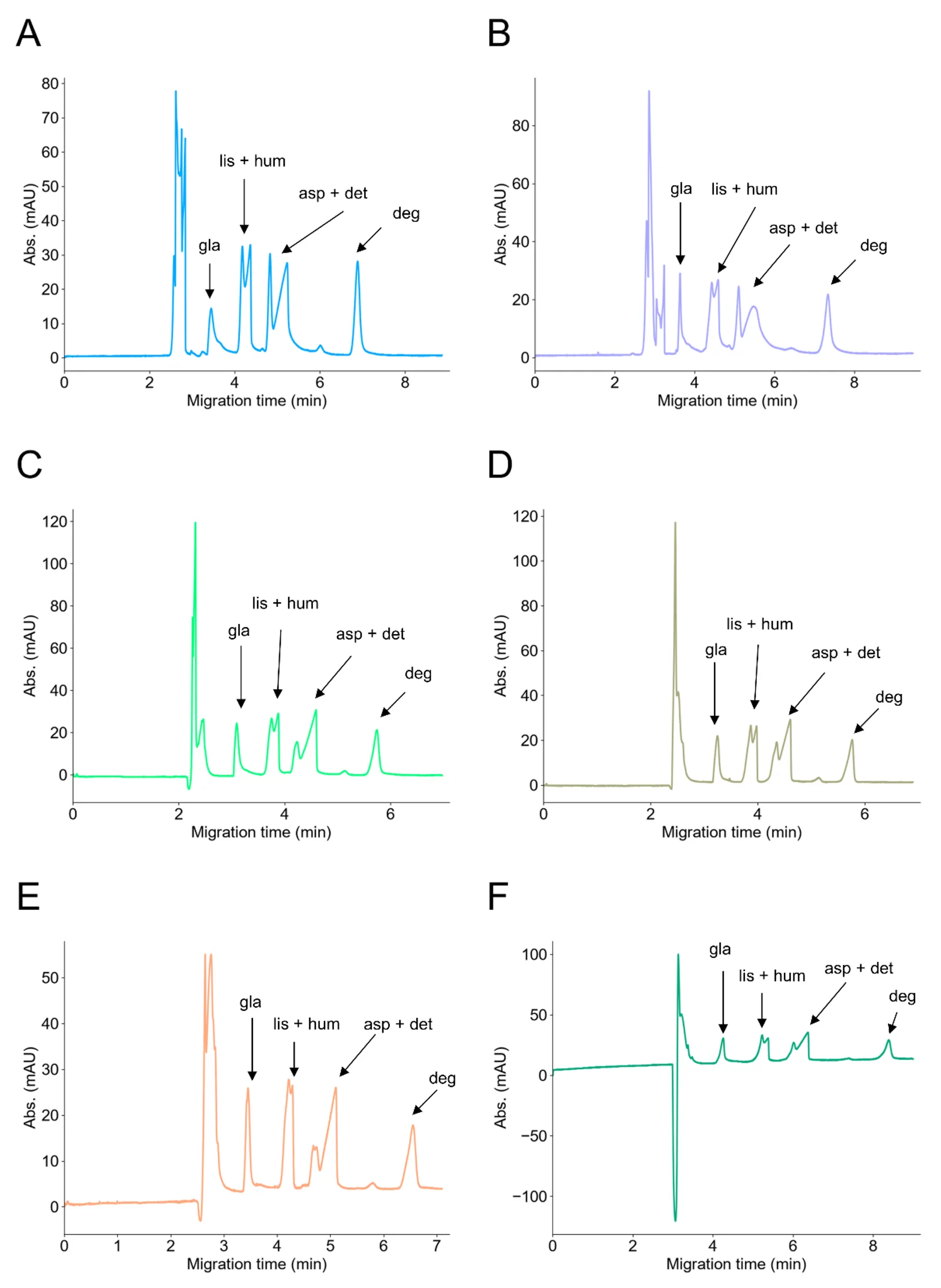
Representative electropherograms obtained using the (PDADMAC/PSS)_2_ SMIL-coated capillary with different background electrolytes: (**A**) 25 mM borate buffer, pH 9.0; (**B**) 50 mM borate buffer, pH 9.0; (**C**) 50 mM ammonium acetate, pH 9.0; (**D**) 25 mM ammonium formate, pH 9.0; (**E**) 50 mM ammonium formate, pH 9.0; (**F**) 500 mM Tris(hydroxymethyl)aminomethane buffer, pH 9.0. Applied voltage: 15 kV; capillary temperature: 25 °C; hydrodynamic injection: 50 mbar × 4 s. Detection at 210 nm.

**Figure 5:**
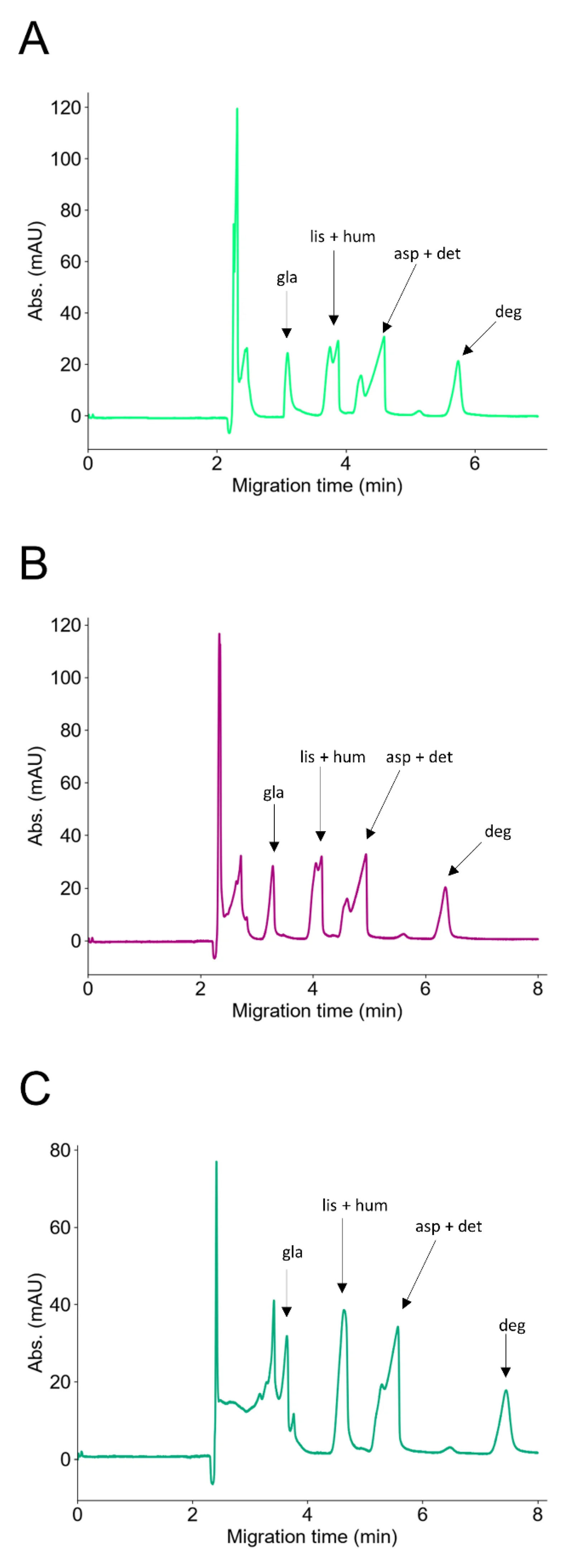
Representative electropherograms obtained using a (PDADMAC/PSS)_2_ SMIL-coated capillary with 50 mM ammonium acetate buffer at different pH values: (**A**) pH 9.0; (**B**) pH 9.5; (**C**) pH 10.0. Other conditions are the same as in Figure 4.

Given these considerations, several basic BGEs were investigated, including 25 and 50 mM borate buffers, 25 and 50 mM ammonium acetate buffers, 25 and 50 mM ammonium formate buffers, and 500 mM Tris, all adjusted to pH 9.0. Ammonium acetate- and ammonium formate-based buffers provided broadly similar separation profiles, while both resulted in superior peak shapes compared with the borate buffers. Based on the overall separation profile, considering both selectivity and peak shape, 50 mM ammonium acetate at pH 9.0 was selected for subsequent optimization (Figure 4). During the subsequent pH screening, pH values of 9.0, 9.5, and 10.0 were investigated using the same 50 mM ammonium acetate BGE. Increasing the pH above 9.0 resulted in a gradual deterioration of the separation and inadequate selectivity for insulin glargine (Figure 5). Therefore, pH 9.0 was selected for further optimization.

#### 2.2.2. CD Screening

**Figure 6:**
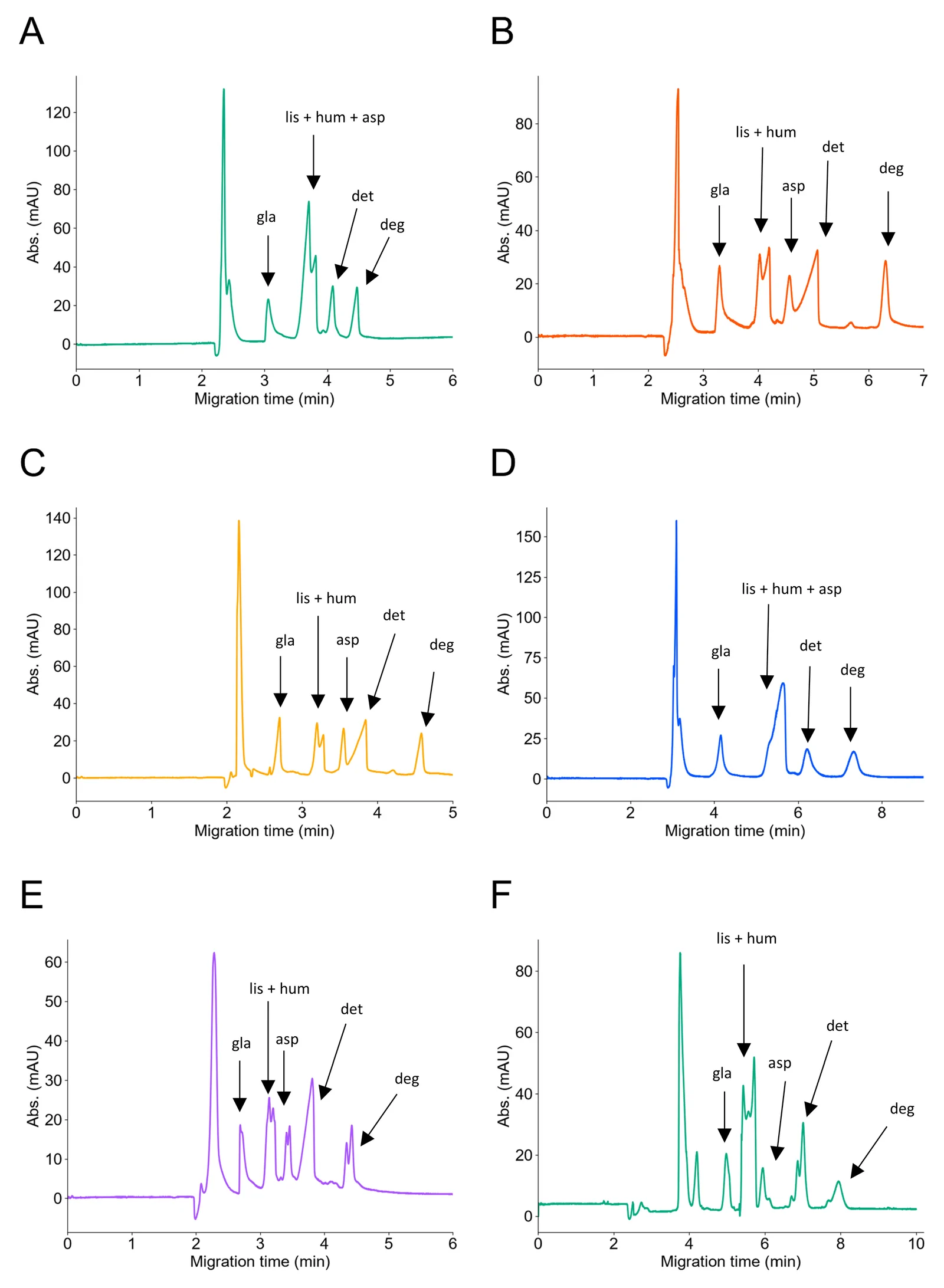
Representative electropherograms obtained using a (PDADMAC/PSS)_2_ SMIL-coated capillary with 50 mM ammonium acetate buffer at pH 9.0 and different cyclodextrin additives: (**A**) α-CD (20 mM); (**B**) CE-γ-CD (2 mM); (**C**) phosphorylated β-CD (2 mM); (**D**) HP-β-CD (5 mM); (**E**) HS-β-CD (2 mM); (**F**) SBE-β-CD DS4 (2 mM).

Following the selection of 50 mM ammonium acetate at pH 9.0, a total of 22 CD derivatives were evaluated as BGE additives (Supplementary Table S2). Representative electropherograms obtained with selected CD derivatives are presented in Figure 6.

Compared with the CD-free background electrolyte, the addition of CDs modified both the migration behavior and the separation selectivity of the insulin species. Compared with the acidic separation system, where the migration order was degludec–detemir–aspart–human insulin–lispro–glargine, the alkaline conditions resulted in the opposite migration sequence: glargine–lispro–human insulin–aspart–detemir–degludec. This reversal reflects the combined effect of the cathode-directed EOF generated by the PSS-terminated SMIL coating and the change in the electrophoretic mobilities of the insulin analogues under alkaline conditions. The investigated CD derivatives did not alter this migration sequence but primarily affected the relative spacing between neighboring peaks.

In the absence of CDs, the electropherogram consisted of three characteristic regions. Insulin glargine migrated as a well-resolved peak, followed by a closely migrating insulin lispro–human insulin peak pair and a second cluster comprising insulin aspart and insulin detemir, whereas insulin degludec remained clearly separated as the last migrating analogue. The addition of the various CD derivatives modified the spacing within these regions to different extents. Depending on the CD derivative, the resolution of one critical peak pair could be improved, whereas only minor changes were observed in other parts of the electropherogram. Although several CD derivatives enhanced the selectivity of the separation, none of the screened CDs provided baseline separation of all six insulin species under the investigated conditions.

Satisfactory peak shapes were not obtained for all analytes. In particular, human insulin and insulin detemir consistently exhibited pronounced peak fronting irrespective of the investigated CD derivative, indicating that additional optimization of the separation conditions was still required.

In contrast to the acidic separation system, the alkaline conditions enabled the application of anionic cyclodextrin derivatives without deterioration of the SMIL coating. Whereas highly negatively charged CDs disrupted the PDADMAC-terminated coating under acidic conditions, the PSS-terminated coating remained stable under the present experimental conditions when low concentrations of anionic CDs were employed. Consequently, in addition to neutral CD derivatives, the screening also included single-isomer anionic CDs (HDMS-α-CD, HDMS-β-CD, ODMS-γ-CD, and HS-β-CD), randomly substituted carboxyalkylated derivatives, and SBE-β-CDs, as well as S-β-CD and P-β-CD.

An interesting concentration-dependent phenomenon was observed for the anionic CD derivatives. At concentrations of approximately 2 mM or higher, several insulin peaks gradually split into multiple components, resulting in increasingly complex electrophoretic profiles. This behavior was consistently observed for all investigated classes of anionic CDs but was not detected in the presence of neutral CD derivatives (see, for example, Figure 6E,F).

Overall, the results demonstrate that the addition of CDs can modulate the electrophoretic selectivity of insulin species under alkaline conditions. Although baseline separation of all six analytes was not achieved during the initial screening, several CD derivatives produced measurable improvements in the resolution of the critical peak pairs, providing a suitable basis for further optimization of the separation system.

To further improve peak shape, the effect of organic modifiers was also investigated at a 10% (*v*/*v*) level. Although the addition of organic solvents resulted in a slight improvement in peak shape for several insulin analogues, this was accompanied by a reduction in separation selectivity. Consequently, the observed improvement in peak shape did not compensate for the loss of resolution, and the use of organic modifiers was not considered advantageous under the investigated conditions. Overall, despite the CD-induced changes in selectivity, the alkaline system did not provide sufficient improvement to achieve baseline separation of all six insulin species.

## 3. Materials and Methods

### 3.1. Chemicals and Reagents

Ammonium acetate, acetonitrile (gradient grade), formic acid (FA, 98% *v*/*v*), sodium chloride, sodium hydroxide, potassium dihydrogen phosphate, and o-phosphoric acid 85% were purchased from VWR (Radnor, PA, USA). Glacial acetic acid, methanol, and ethanol were obtained from Chemical Company (Iasi, Romania). Hydrochloric acid 36% was purchased from Scharlau Chemie (Barcelona, Spain). Tris(hydroxymethyl)aminomethane (Tris) was obtained from Sigma-Aldrich (St. Louis, MO, USA). Ultrapure water was prepared using a Barnstead Nanopure Diamond water purification system (Boston, MA, USA). As coating agents, poly(diallyldimethylammonium chloride) (PDADMAC, 20% (*w*/*w*) in water, Mw 400,000–500,000, cat. no. 409030) and poly(sodium 4-styrenesulfonate) (PSS, Mw ~1,000,000, cat. no. 434574) were purchased from Sigma-Aldrich. The cyclodextrins (CDs) applied in this study were all products of Cyclolab Ltd. (Budapest, Hungary). Coating solutions were prepared from each polymer at 0.3% (*w*/*v*) in 0.5 M NaCl.

### 3.2. Human Insulin and Its Analogues

For the analysis, insulin aspart (asp, NovoRapid; 100 U/mL, 3.5 mg/mL), insulin degludec (deg, Tresiba; 100 U/mL, 3.66 mg/mL), and insulin detemir (det, Levemir; 100 U/mL, 14.2 mg/mL) were obtained from Novo Nordisk (Bagsværd, Denmark). Insulin glargine (gla, Toujeo SoloStar^®^; 300 U/mL, 10.91 mg/mL) was obtained from Sanofi Aventis (Paris, France), and insulin lispro (lis, Humalog^®^; 100 U/mL, 3.5 mg/mL) from Eli Lilly (Indianapolis, IN, USA). Recombinant human insulin (cat. no. I2643) was purchased from Sigma-Aldrich (St. Louis, MO, USA) and dissolved in 0.1 M hydrochloric acid to prepare a 3.5 mg/mL stock solution. Prior to analysis, all solutions were filtered through 0.45 μm membrane filters. Stock solutions were stored at 4 °C. For the preparation of the final sample mixture, 15 µL each of insulin detemir and insulin glargine were mixed with 25 µL each of insulin lispro, insulin aspart, insulin degludec, and human insulin. Subsequently, 25 µL of 1 M formic acid and 400 µL of deionized water were added.

### 3.3. Instrumentation and CE Parameters

Separation experiments were conducted using an Agilent G1600 CE (Agilent Technologies, Waldbronn, Germany), equipped with a photodiode array detector. ChemStation software (Rev. B 04.03) was employed for data acquisition and analysis. Untreated fused-silica capillaries of 50 µm I.D. were purchased from Polymicro (Phoenix, AZ, USA).

Unless stated otherwise, the capillary temperature was 25 °C, and samples were injected hydrodynamically at 50 mbar for 4 s.

### 3.4. Preparation and Use of SMIL-Coated Capillaries

Multilayer dynamic capillary coating was performed using a PrinCEC-760 (Prince Technologies, Emmen, The Netherlands) CE apparatus, capable of delivering 3 bar flushes without the use of external pressure. Prior to coating, the 70 cm long fused-silica capillaries (without a detection window) were flushed with 1 M NaOH for 20 min, followed by ultrapure water for 3 min at 2.3 bar.

A (PDADMAC/PSS)_2_ multilayer coating was prepared by sequentially flushing the capillary with 0.3% (*w*/*v*) PDADMAC in 0.5 M NaCl for 20 min, ultrapure water for 3 min, 0.3% (*w*/*v*) PSS in 0.5 M NaCl for 20 min, and ultrapure water for 3 min. The same PDADMAC/PSS deposition sequence was subsequently repeated, resulting in the four-layer (PDADMAC/PSS)_2_ coating. All flushes were performed at 2.3 bar. This resulted in a PSS-terminated (anionic) coating.

The five-layer (PDADMAC/PSS)_2.5_ coating was prepared by applying an additional PDADMAC layer after the second PSS deposition, yielding a PDADMAC-terminated coating with a cationic outer layer.

Following the coating procedure, the 70 cm capillaries were cut to a total length of 33 cm, yielding two coated capillaries from each preparation. A detection window was created at 8.5 cm from the outlet end, resulting in an effective capillary length of 24.5 cm. Before first use, the capillaries were preconditioned with the corresponding BGE for 20 min in the Agilent CE instrument.

The five-layer (PDADMAC/PSS)_2.5_ coating was used for separations under acidic conditions, whereas the four-layer (PDADMAC/PSS)_2_ coating was used under alkaline conditions, as described in the Section 2.

## 4. Conclusions

In the present study, the effect of CD derivatives on the CE separation of human insulin and five therapeutic insulin analogues was systematically investigated using two differently terminated SMIL-coated capillaries operated under acidic and alkaline conditions. The results demonstrated that the addition of CDs can modulate the electrophoretic selectivity of these structurally closely related insulin species, although complete baseline separation of all analytes could not be achieved under a single set of experimental conditions.

The two coating strategies exhibited markedly different separation characteristics. Under acidic conditions, neutral CD derivatives improved separation selectivity, whereas highly charged anionic CD derivatives destabilized the PDADMAC-terminated coating and therefore could not be employed. In contrast, the PSS-terminated coating remained stable under alkaline conditions, allowing the investigation of both neutral and anionic CD derivatives. The alkaline separation system also exhibited a complete reversal of the migration order of the insulin species, reflecting the combined effects of the cathode-directed EOF and their altered electrophoretic mobilities under alkaline conditions. Furthermore, anionic CD derivatives produced a reproducible concentration-dependent peak-splitting phenomenon, the origin of which remains to be elucidated.

The effect of organic modifiers was strongly dependent on the separation system. Under acidic conditions, methanol and ethanol improved the resolution of the degludec/detemir pair and reduced peak fronting, with ethanol providing the most favorable overall separation performance. In contrast, under alkaline conditions, the slight improvement in peak shape obtained with organic modifiers was accompanied by reduced separation selectivity. These findings further demonstrate that the effects of both CD derivatives and organic modifiers depend strongly on the coating and BGE conditions employed.

To the best of our knowledge, this is the first systematic study evaluating a broad range of neutral and charged CD derivatives for the CE separation of therapeutic insulin analogues using SMIL-coated capillaries. Beyond the analytical application presented here, the results provide further evidence that CDs can serve as effective selectivity-modifying additives for peptide separations by CE. While the use of CDs is well established for the separation of small molecules, their application to peptides has been explored to a considerably lesser extent. The present findings expand current knowledge in this area and demonstrate the potential of combining appropriate CD derivatives with complementary SMIL coating strategies to modulate selectivity in the CE separation of structurally closely related insulin species.

## Data Availability

All relevant data are contained within the article and the Supplementary Materials.

## Supplementary Materials

The following supporting information can be downloaded at https://www.mdpi.com/article/10.3390/molecules31193578/s1, Table S1: CD derivatives used with the (PDADMAC/PSS)_2.5_ coated capillaries; Table S2: CD derivatives used with the (PDADMAC/PSS)_2_ coated capillaries; Figure S1. Representative electropherograms illustrating the effect of increasing CD concentration on the separation of insulin species, obtained using a (PDADMAC/PSS)_2.5_ SMIL-coated capillary. BGE: 1.5 M acetic acid and CD additives as shown on the figure. Applied voltage: −30 kV; Capillary temperature: 25 °C; Hydrodynamic injection: 50 mbar × 4 s. Detection at 210 nm.
