# Supplementary material for: Effect of Cyclodextrin Complexation on the Separation of Insulin Analogues in Capillary Electrophoresis Using Dynamically Coated Capillaries
Source: Molecules. 2026 Oct 8;31(19):3578. doi: 10.3390/molecules31193578 (PMC13667192; doi:10.3390/molecules31193578)
Supplement: Supplementary file 1 [file molecules-31-03578-s001.zip › molecules-4506157-supplementary.pdf]

Name	Concentration (mM)	Abbreviation	DS	Source
α-cyclodextrin	5-10-20	α-CD	-	Cyclolab
β-cyclodextrin	2,5-5-10	β-CD	-	Cyclolab
γ-cyclodextrin	5-10-20	γ-CD	-	Cyclolab
Hydroxypropyl-β-CD	5-10-20	HP-β-CD	4,5	Cyclolab
Randomly Methylated β-Cyclodextrin	5-10-20	RAMEB	12	Cyclolab
Heptakis(2,6-di-O-methyl)-β-cyclodextrin	5-10-20	DIMEB	14	Cyclolab
Succinyl-β-cyclodextrin	5-10-20	Succ-β-CD	3,5	Cyclolab
Succinyl-hydroxypropyl-β-cyclodextrin	5-10-20	SuccHP-β-CD		Sigma Aldrich
Acetyl-β-cyclodextrin	5-10-20	Ac-β-CD	7	Cyclolab
Carboxymethyl-α-cyclodextrin sodium salt	0,5-1-2	CM-α-CD	3,5	Cyclolab
Carboxymethyl-β-cyclodextrin sodium salt		CM-β-CD	3,5	Cyclolab
Carboxymethyl-γ-cyclodextrin sodium salt	0,5-1-2	CM-γ-CD	3,5	Cyclolab
Carboxyethyl-α-cyclodextrin	0,5-1-2	CE-α-CD	3	Cyclolab
Carboxyethyl-β-cyclodextrin	0,5-1-2	CE-β-CD	3	Cyclolab
Carboxyethyl-γ-cyclodextrin	0,5-1-2	CE-γ-CD	3	Cyclolab

Table S1. CD derivatives used with the $(PDADMAC/PSS)_{2.5}$-coated capillaries

Table S2. CD derivatives used with the $(PDADMAC/PSS)_2$-coated capillaries

**Name**	**Concentration (mM)**	**Abbreviation**	**DS**	**Source**
α-cyclodextrin	2,5-5-10-20	α-CD	-	Cyclolab
β-cyclodextrin	2,5-5-10	β-CD	-	Cyclolab
γ-cyclodextrin	5-10-20	γ-CD	-	Cyclolab
Hydroxypropyl-β-CD	5-10-20	HP-β-CD	4,5	Cyclolab
Randomly Methylated β-Cyclodextrin	5-10-20	RAMEB	12	Cyclolab
Heptakis(2,6-di-O-methyl)-β-cyclodextrin	5-10-20	DIMEB	14	Cyclolab
Succinyl-β-cyclodextrin	0,5-1-2	Succ-β-CD	3,5	Cyclolab
Succinyl-hydroxypropyl-β-cyclodextrin	5-10-20	SuccHP-β-CD		Sigma Aldrich
Sulfobutylated-β-cyclodextrin sodium salt	0,5-1-2	SBE- β-CD	4	Cyclolab
Sulfobutylated-β-cyclodextrin sodium salt	0,5-1-2	SBE-β-CD	6	Cyclolab
Sulfated-β-cyclodextrin	0,5-1-2	S-β-CD	12-15	Sigma Aldrich
Hexakis-(2,3-di-O-methyl-6-O-sulfo)-α-CD	0,5-1-2	HDMS-α-CD	18	Cyclolab
Heptakis-(2,3-di-O-methyl-6-O-sulfo)-β-CD	0,5-1-2	HDMS-β-CD	21	Cyclolab
Octakis-(2,3-di-O-methyl-6-O-sulfo)-γ-CD	0,5-1-2	ODMS-γ-CD	24	Cyclolab
Heptakis-(6-O-sulfo)-β-cyclodextrin	0,5-1-2	HS-β-CD	7	Cyclolab
Carboxymethyl-α-cyclodextrin	0,5-1-2	CM-α-CD	3,5	Cyclolab
Carboxymethyl-β-cyclodextrin	0,5-1-2	CM-β-CD	3,5	Cyclolab

Carboxymethyl-γ-cyclodextrin	0,5-1-2	CM-γ-CD	3,5	Cyclolab
Carboxyethyl-α-cyclodextrin	0,5-1-2	CE-α-CD	3	Cyclolab
Carboxyethyl-β-cyclodextrin	0,5-1-2	CE-β-CD	3	Cyclolab
Carboxyethyl-γ-cyclodextrin	0,5-1-2	CE-γ-CD	3	Cyclolab
β-cyclodextrin phosphate	0,5-1-2	P-β-CD	2-6	Cyclolab

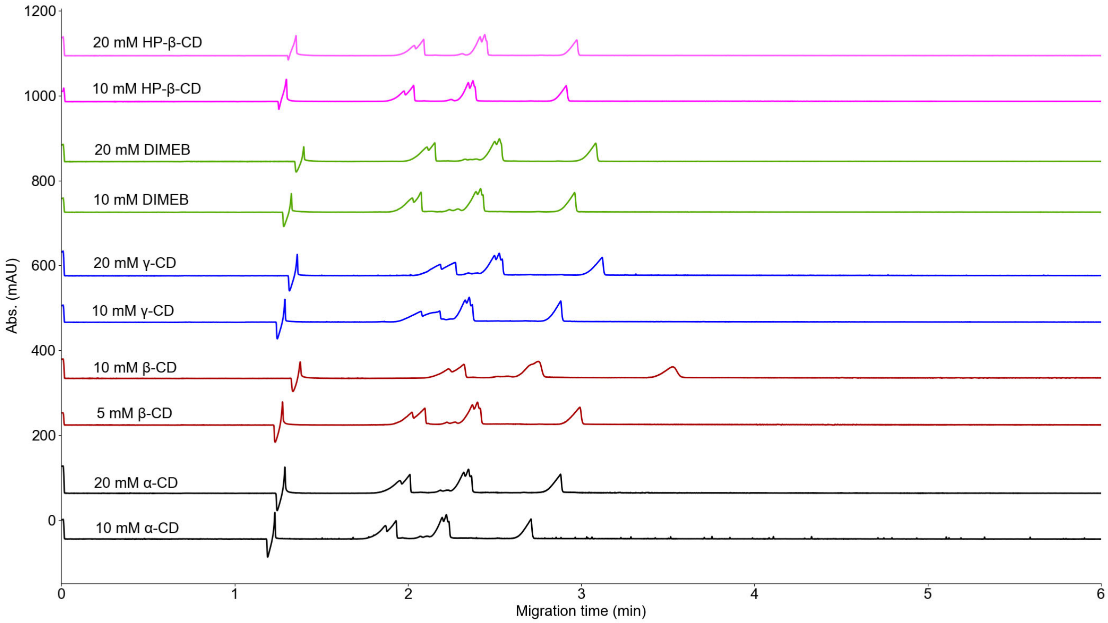

Figure S1. Representative electropherograms illustrating the effect of increasing CD concentration on the separation of insulin species, obtained using a $(PDADMAC/PSS)_{2.5}$ SMIL-coated capillary. BGE: 1.5 M acetic acid and CD additives as shown on the figure. Applied voltage: -30 kV; Capillary temperature: 25 °C; Hydrodynamic injection: 50 mbar x 4 s. Detection at 210 nm.
